# Clinician perspectives on the process of change in an adolescent eating disorder Day Program

**DOI:** 10.1186/s40337-025-01457-x

**Published:** 2025-12-29

**Authors:** Nicola White, Lisa Dawson, Deborah Mitchison, Andrew Wallis

**Affiliations:** 1 Body Image and Eating Academic Network, Clinical Psychology, Faculty of Health, University of Technology Sydney, Sydney, Australia; 2 Eating Disorder Intensive Program for Adolescents, Department of Psychological Medicine, Sydney Children’s Hospitals Network, Sydney, Australia; 3 Eating Disorder Service Network Lead, Sydney Children’s Hospitals Network, Department of Psychological Medicine, Sydney, Australia

**Keywords:** Anorexia nervosa, Day program, Change mechanisms, Qualitative analysis, Clinician perspective

## Abstract

**Background:**

Despite suggestions that Day Programs can be efficacious, little is understood about which mechanisms create positive change for young people and their family in this setting. The aim of this study was to explore clinician perspectives on how change occurs in an adolescent eating disorder Day Program in Australia.

**Methods:**

Twelve clinicians working as a team on an adolescent Day Program participated in semi-structured qualitative focus groups. Focus groups explored the clinicians’ perspectives on change processes in the Day Program treatment model and the impact on young people and their families. Data generated from each focus group was analysed using reflexive thematic analysis.

**Results:**

The structure, predictability and intensity of the Day Program along with tailored and responsive treatment was perceived as leading to a main theme of structural containment. Additionally, the combination of a strong relational treatment focus with team mutuality was perceived as leading to a second main theme of relational containment. The combination of structural and relational containment for families was perceived by clinicians as the leading mechanisms in supporting parental effectiveness in treatment and the settling and engaging of the young people. Levels of containment were also understood to create safety for all (parents, young people, and clinicians) providing a wraparound approach allowing effective treatment to be delivered.

**Conclusions:**

The findings of this study speak to the importance of safety as a foundation for working with families needing higher levels of care. Treatment approaches that are both structurally and relationally containing might be needed for families requiring more intensive care. Findings also highlight the importance of the clinical team working cohesively and the potential need for clinicians/services to consider how to create a therapeutically supportive environment that maximises the effectiveness of treatment.

## Abstract (plain language summary)

While there is evidence that Day Program treatment is helpful, little is understood about what creates positive change for families in this setting. This study aimed to explore how clinicians believe change to occur during Day Program treatment. For this aim, twelve clinicians participated in semi-structured qualitative focus groups of 60 to 90 min in the Day Program setting. Data from each focus group was analysed using reflexive thematic analysis, a widely used method for analysing this type of data.

The Day Program’s structure, predictability, intensity, and tailored and responsive treatment was understood as leading to a main theme of structural containment. Additionally, the combination of team mutuality and building strong relationships was understood as leading to a second main theme of relational containment. It was understood that clinicians perceived these two main themes – structural and relational containment - as the main factors in supporting positive change for young people and their families.

The present study highlights the importance of safety when working with families who need more intensive care. Families who attend Day Program may benefit from treatment that provides both clear structure and strong relational support. This study also highlights the importance of clinical teams working together and for services to create a supportive environment that enhances effective treatment for families.

## Background

For a significant minority of young people with anorexia nervosa (AN), standard outpatient and/or inpatient treatment models are insufficient to support recovery from an eating disorder [1–3]. To address the needs of young people with more complex treatment needs, many services have looked to offer intensive outpatient treatment models, such as Day Programs [4–7]. Day Program treatment models can vary significantly, and there is no one model across services for young people with AN [8]. For example, some Day Programs utilise a Family-Based Therapy (FBT) model or a cognitive-behavioural model (CBT), or a mixture of both treatments [9, 10]. In general, however, a Day Program will consist of (1) increased treatment intensity compared to outpatient care, with young people typically attending multiple days per week for several hours per day [9, 11]; and (2) young people leaving the facility to return home at the end of the day, in contrast to inpatient treatment [5]. In turn, Day Programs can function as a step-up from outpatient care and a step-down from inpatient treatment [12, 13]. One systematic review has shown that Day Programs, generally (i.e., across age and treatment models of focus), have been associated with physical and psychological change including weight gain, improvements in comorbid psychopathologies (e.g., depression, anxiety and worry symptoms), and improvements in psychosocial functioning (e.g., emotion regulation, cognitive flexibility and attachment relationships; [12]).

While empirical research on Day Program treatment is growing, relatively little is known about designing effective Day Programs [12]. Service design of Day Programs, while empirically informed, is also driven by clinical wisdom and experience. Little is known about the possible mechanisms for how positive changes in a Day Program treatment setting might occur [14]. Understanding mechanisms for change that lead to improvements in eating disorder symptoms and broader relational and psychological changes for young people and their family is important to understand, to enable improvement to treatment interventions, as well as understanding the unique treatment features of a Day Program model and how this impacts care.

One previous qualitative study investigating the experiences of young people and their families found that containment of the AN and the structure of Day Program were instrumental in creating change, including reducing AN symptomology and improving comorbid psychopathologies for the young people [5]. Containment in this context refers to the process of managing or controlling the symptoms associated with AN (e.g., predictability with mealtimes; [15]), while structure typically refers to a strict schedule that the young people and parents are expected to follow during the program (e.g., expectations surrounding eating) [16]. Containment and program structure have been found to be useful in this setting, as they provide families with clear expectations, which in turn creates a secure base and settles the family [5, 16]. These perspectives from young people and their families provide valuable insights into what aspects of a Day Program are helpful, however, additional perspectives are needed to gain a fuller picture of what creates change in this setting, with a particular focus on service design and service delivery.

One perspective that has been largely overlooked includes the experience of clinical staff at a Day Program [9, 16, 17]. Exploring clinician perspectives would likely provide unique insights given staff have firsthand experience of the family’s behaviours, challenges and development; and have observed patterns across the many families who have accessed a Day Program over time; and, most importantly are involved in treatment design and delivery. Indeed, there is little in the literature at large regarding how clinical teams understand and articulate their philosophy of change and clinical thinking regarding how change occurs in treatment settings. Thus far, only two qualitative studies have explored clinicians’ perspectives on their experiences in specialist eating disorder intensive treatment settings [16, 17]. These studies identified processes such as flexibility, carer burden, and intensive support, and while informative, either did not focus on adolescent Day Programs, or reported on *what* their experience was like working in these settings (e.g., working with limited resources), and not *how* change occurred for the patients.

This paper aims to use the rigor of qualitative research methods to explore and overt the clinical thinking of a team of specialist eating disorder clinicians to investigate their understanding of how positive treatment changes occur within an adolescent eating disorder Day Program at the Sydney Children’s Hospital Network (SCHN). This research complements existing data on the perceived change processes reported from the perspectives of young people and their families and provides valuable insights for refining content and processes to target in treatment into the future, with the aim of improving treatment outcomes.

## Method

### Day program treatment description and study site

The participating study site was the Eating Disorder Intensive Program for Adolescents (EDIPA) which is part of the Eating Disorder Service at Sydney Children’s Hospital’s Network (SCHN). The program was developed to meet the needs of patients and families for whom standard outpatient care was not sufficient to support recovery, or for families who needed a step down from inpatient admission owing to the severity of their illness.

The Day Program at EDIPA is a brief but intensive outpatient program where young people (between the ages of 12 to 18) attend from Monday to Friday from 9am to 4pm for an average duration of 14 weeks. Admission into EDIPA requires young people to meet a level of complexity where they are not progressing in standard outpatient treatment, however their needs are not so severe that they require inpatient care. Treatment is initially full time and then transitions to part time attendance as treatment progresses and young people gradually reconnect to adolescent life. Discharge from EDIPA is appropriate when outpatient treatment is sufficient to meet the young person and family’s needs and typically occurs when a young person has reached physical recovery, there has been a reduction in eating disorder symptomology, and an increase general functioning, such as capacity to return to school. The program has a family therapy model of care where family involvement plays a central role in treatment. The program utilises FBT principles as well as drawing on systemic family therapy, attachment-based therapies and dialogical practices. The entire multi-disciplinary team participate in weekly reflective, dialogical group supervision. In addition, regular team training days are held where clinicians discuss the program’s model of care and the processes of change. There is a focus on physical, psychological, and functional recovery from an eating disorder, and treatment has a strong relational focus and aims to be responsive and tailored to the needs of each family. The program includes therapeutic meal support, a group-based therapeutic program (including CBT and Dialectical Behaviour Therapy-based groups), psychiatry and medical treatment, attending an in-house school program, parent groups, and individual and family therapy. Parents attend two parent groups per week, a multi-family morning tea where in vivo meal support is provided, and weekly family therapy. Daily check-ins or ad-hoc meal support with parents is provided as needed. Treatment is delivered in an inter-disciplinary model of care and in partnership with the parents, with an emphasis on both behavioural changes and the strengthening of family relationships. At the time of writing this manuscript, an interdisciplinary team of 12 clinical staff worked at EDIPA (see more below under Participants).

### Recruitment

All participants were recruited from EDIPA and informed of the study via email from a member of the broader SCHN eating disorders service team who was not an EDIPA team member or line manager of any participants, nor a researcher on the study, and from a different hospital campus. If an individual was interested in participating, the staff member would notify a member of the research team, first author, NW, who would then provide participants with a participant information sheet and consent form.

### Participants

Of the 12 clinical staff approached to participate, all consented and took part in the present study. These included four clinical psychologists, one clinical psychology registrar, one assistant in nursing, one registered nurse, one psychiatry registrar, one psychiatrist, one paediatrician, one clinical social worker, and one dietitian. Each clinician’s time spent working at EDIPA spanned from five months to 10 years, since the program commenced.

### Design

Six qualitative focus groups of 60 to 90 min were conducted either face-to-face, or via video-call on Microsoft Teams, with the first author, NW. The mode of the focus group was based on participant preference. Each focus group included two clinical staff members and were conducted throughout August 2024. Participants were interviewed with clinicians with a similar clinical background (e.g., clinical psychologist with a clinical psychologist), where possible. A similar clinical background for the groups was chosen to help create an interview space where the clinicians could exchange ideas and draw upon their shared professional experiences. All focus groups were semi-structured and followed the same sample guide, which included questions about participants’ perception of what creates change among young people and families in the program. Prompts and guidance were also provided if content relevant to the study was not spontaneously raised by the participants. These included prompts to explore the structure of EDIPA, relationships with families, and specific parts of EDIPA. All focus groups were audio-recorded using a voice recording device and transcribed verbatim.

### Data analysis

Data generated from each focus group were analysed using Reflexive Thematic Analysis [18]. This method of analysis was chosen to enhance transparency and credibility of the findings by acknowledging the authors’ subjectivity throughout the analysis. To assist the analysis, NW used the qualitative data analysis software, NVivo 14. A critical realist framework was adopted when analysing the data which meant that experiences and meaning of the data were considered subjective and influenced by social and cultural context [19, 20]. NW approached the data with prior knowledge about Day Programs and its change mechanisms from previous qualitative data exploring the experiences of young people and their families [4, 5]. Given this approach, analysis of the data was deductive and semantic, however this was flexible. For example, at times participants in the current study raised themes that had not been widely explored in the literature and in the context of Day Program. Consequently, despite this deductive approach, the data was not restricted to a particular theoretical lens and still provided a rich description of the overall dataset.

One of the themes that was raised by participants that had not been widely reported on in previous research was relationships between young people in the program. During the data analysis process, the researchers became aware that the participants spoke from one perspective and had not been asked in the focus groups to explore this from other perspectives. To address this imbalance in the data collection process, the participants were emailed this question after the data collection period and invited to respond. These responses were then included in the final data analysis.

**Fig. 1:**
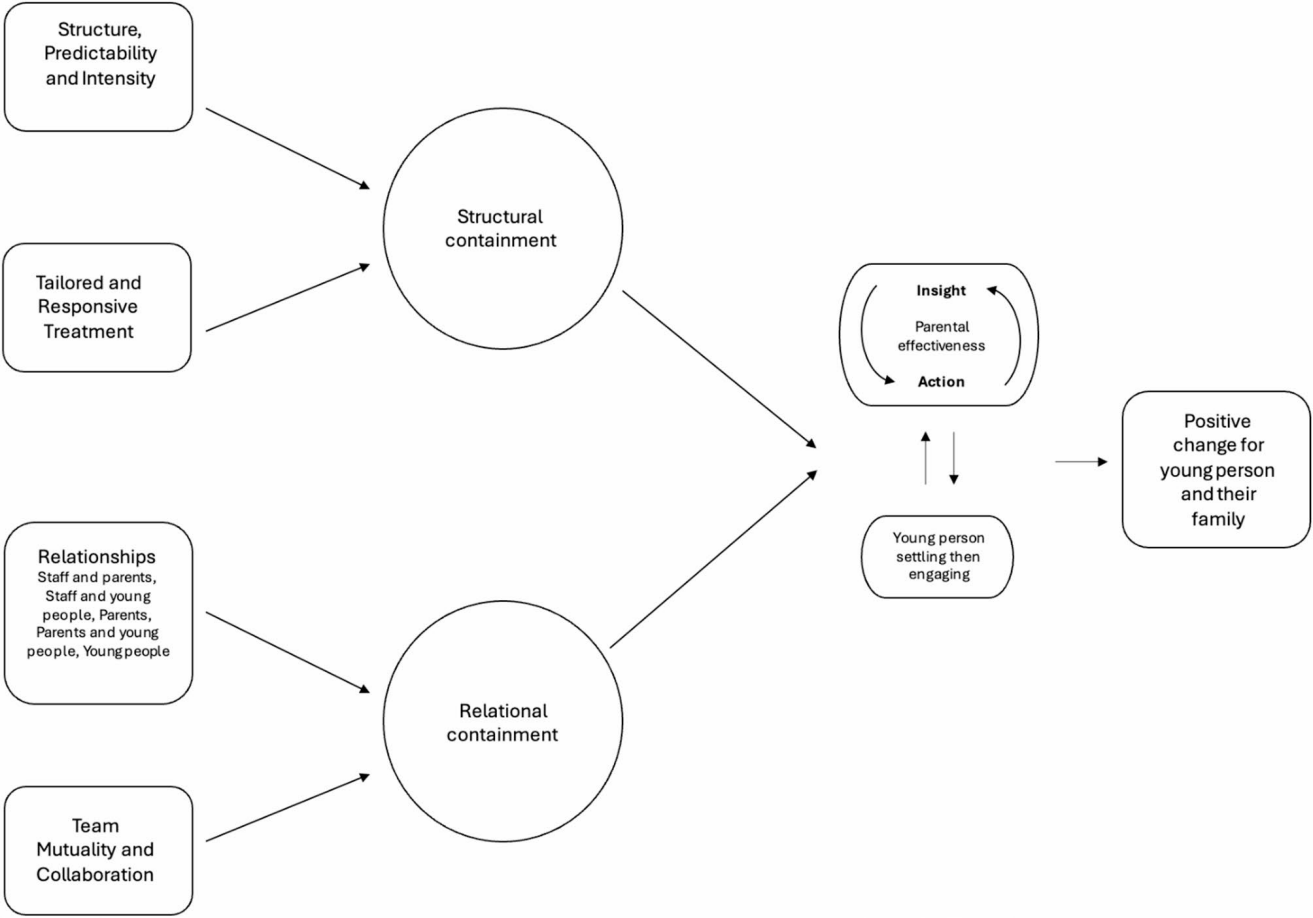
Perceived change process in an adolescent eating disorder Day Program from the clinician perspective

The six-phase process of reflexive thematic analysis [18] were followed. Initially, the first author familiarised herself with the data (step 1) by reading through each transcript several times. Codes were then produced (step 2) and themes generated (step 3). These themes were then reviewed (step 4) by Coordinating Principal Investigator (CPI), LD, and DM, to ensure they reflected accurate descriptions of the data and answered the research question. Thereafter, themes were named and finalised (step 5), and the manuscript was written (step 6). The change process model (see Fig. 1 below) was developed by NW, LD and AW and reviewed and finalised by all authors.

Throughout the analysis, various measures to increase the rigor of the present study were employed. These included the first author keeping a journal of memo writing to document and reflect on her ideas throughout the research process, exporting codes into a Microsoft Word document to further sort and organise, keeping an audit trail, and member checking to increase rigor of the findings.

### Member checking

Member checking was completed in line with the recommended guidelines to increase the rigor of the study and the trustworthiness of the results [21]. After themes were generated from the data, all participants were sent a short summary of the results. Thereafter, participants could discuss via email whether the results reflected their experiences and add or amend anything to the ideas presented. In all, 10/12 (83.3%) participants responded and confirmed that the results were consistent with their experiences and did not suggest any amendments to the results. The remaining two participants did not respond.

### Reflexivity statement

In addition to being a student researcher at the time of data collection and analysis, NW was a clinical psychology student and completed her final clinical placement at EDIPA between late July and December 2024. LD and AW are also both participants and principal investigators of the present study. LD is a clinical psychologist and team leader at EDIPA and AW is a clinical social worker and senior member of the EDIPA team. As such, their professional backgrounds influenced the lens through which the data were analysed. To manage this influence, data was initially reviewed by NW and DM to establish an initial foundation for the findings before further analysis. In addition, the research team engaged in regular reflexive discussions to check for assumptions throughout the coding process and writing of the manuscript.

## Results

Two main themes of (1) structural containment and (2) relational containment were identified, organised into two pathways, and understood as working in combination to facilitate and activate essential treatment processes. The first pathway depicts the combined effects of Structure, Predictability and Intensity, along with Tailored and Responsive Treatment, leading to the overarching theme of Structural Containment; which was defined as families feeling safe and supported by the external or operational aspects of EDIPA. The second pathway depicts a combination of Relationships, and Team Mutuality and Collaboration creating an overarching theme of Relational Containment. Relational containment was defined as both families and the clinical team experiencing a sense of social connection and relational safety within EDIPA. Relational safety refers to a sense of psychological and emotional security that is experienced within the therapeutic relationships clients have with clinicians and peers in the program.The combination of structural and relational containment was perceived to activate two essential treatment processes, resulting in a bi-directional process of (1) enhanced parental effectiveness through increased insight and treatment action, which can (2) emotionally and behaviourally settle and then engage the young person. In turn, the settling of the young person allowed for more effective parenting and enhanced capacity for parents to enact the treatment principles in an attuned way. This process was thought to facilitate positive change for the young person and their family (see Fig. 1).

### 1. Structural containment

#### Theme 1a: structure, predictability and intensity

Participants described that due to the severity of the AN among young people at EDIPA, families who attend the program typically present with high levels of anxiety, both in the treatment setting and at home. The clinicians frequently expressed that the structure of EDIPA created clear expectations for the parents and young people to follow. Structure was understood as a schedule in which every moment at EDIPA is dedicated to a particular activity for the young people and parents (e.g., morning tea, school, therapy group etc.).

I think often when families come to us, it can be pretty chaotic. Everyone [is] sort of in a state of like high anxiety and high emotions. And I guess part of being here and part of the structure that creates containment is just sort of knowing when you need to be somewhere and what’s expected of you.

Predictability was seen as arising from the structure and intensity of EDIPA in which the days and weeks when a young person and their family are on program look very similar. The clinicians expressed that this predictability allowed the families to better manage their anxiety because they knew what was expected of them.

…So, the more we can have predictability across the week around expectations, what the days look like, rules, consistency across families for like what those expectations and rules are, the more people can just settle in.

Clinicians often expressed the intensity of the EDIPA program as a needed step up for when a young person required a higher level of care. The move to attending EDIPA five days per week rather than attending outpatient care for around one hour a week was thought to facilitate change in AN symptomology and also understood as placing pressures on parents, albeit necessarily.

I guess the level of intensity and the strength of the illness requires a high level of parental participation and because…the parents are such an important resource for the young person throughout treatment, but [that] obviously then means you need the parent involved and which depending on sort of the work set up and pressure in that way that can be a hard thing.

#### Theme 1b: tailored and responsive treatment

While EDIPA is heavily structured, clinicians spoke of the importance of tailoring treatment within the program to ensure each family’s needs and treatment goals were met. Tailored and responsive treatment was understood as adapting treatment to the individual differences present amongst each family.

There’s some things that are the same for everybody who comes to day program and there are some things that are very like specific and bespoke to the needs of that particular family. And so, there’s also a lot of thinking that happens within the team and with families about what needs to be different in a particular family and how can we use the structure of the day program to meet those needs.

### Structural containment

There was a sense that Structure, Predictability, Intensity and Tailored and Responsive Treatment combined to create Structural Containment. Structural Containment was understood as the external, or operational factors surrounding the program that are supporting the families. The structural containment that the program provides was understood as minimising parental anxiety, effectively supporting parents to settle and engage their young person and create change in their family.

…being in such a sort of structured program where there’s supports across the day and across sort of a week in general, and it allows parents to sort of feel less anxious in their position, which then in turn sort of filters down towards like their ability to be able to do what they need to do to refeed their child.

In fact, clinicians expressed that poor structural containment strengthened the AN, ultimately maintaining the disorder, whereas optimal containment allowed the young people and parents to make changes regarding eating meals, sitting with distress or having difficult conversations with each other and clinicians.

…because we know anorexia is very good at splitting, you know if there is inconsistency and there’s a low structure, it’s gonna, it’s gonna pounce on the idea of spreading because, you know, spreading is something that anorexia thrives on.

I think you can do hard things [eating a meal, sitting with distress, having difficult conversations etc] and do things differently when you feel safe. So, in safety, like [there] is a bit of a strength to like do a hard thing, but there’s a bravery or like a knowing that there’s something around you when you’re doing something hard that will like, hold or catch you.

Interestingly, while the structural containment of EDIPA was understood as a key factor in the perceived change process, some clinicians spoke of how this process can become unhelpful and in fact prevent change when an admission is too long. This is because families can become reliant on the service, ultimately delaying the transition from EDIPA back to community life.

Sometimes when the admissions go too long and we miss those windows for the change, then sometimes that can create sort of an unhelpful sort of dynamic here in which like the mechanisms that were helping change initially then become sort of maintaining things the same and preventing change or creating barriers to sort of the next bit of change.

When parents and young people sort of feel safe and contained here, that can often prevent them from doing that next step of getting back into normal life….

## 2. Relational containment

### Theme 2a: relationships

#### Relationships between staff and parents

Relationships between the staff and parents were described as a *“partnership”* and frequently mentioned as a mechanism for creating and preventing change. On one hand, when a strong therapeutic relationship was present between the staff and parents change was possible because the parents trusted the staff, felt empowered to make changes, and were comfortable sharing their concerns about treatment and their young person’s needs.

But seemingly I think something happens at the beginning when you’re forming relationships with the parents and you’re in partnership with them where they see things starting to shift and they build trust with you, then they lean on you more, I think that emboldens them more to make more changes.

However, if there was a rupture in this relationship, change was prevented because either the trust was broken, or families were not heard by clinicians.

*“Probably like a lack of trust between families and the team*,* yeah that could be something that gets in the way. So*,* if like families are not quite on board or are not quite feeling understood or heard by the team. That might sort of*,* to their minds*,* like maybe not feel supported in what they’re doing or understood.”.*

This partnership was also expressed in the context of clinicians modelling firmness and kindness with the aim that parents start embodying those behaviours to facilitate change in their young person.

…the team are kind of like embodying like kind of meta parents for everyone…I guess in a way of kind of modelling and holding within themselves a kind and loving action-oriented relational stance that creates containment and allows people to do something that’s really hard in a way that they haven’t been able to do it before.

#### Relationships between staff and young people

The relationship between the staff and young people was described as *“firm but loving”* and frequently expressed as a mechanism for creating change. For instance, if the clinicians approached the AN from a strictly firm lens change would not occur because young people would not feel comfortable.

I don’t think you can treat people if you don’t know about them on a bit of a personal level and you just treat them like a number. Like, that’s not going to elicit change because they won’t feel comfortable, and they need to feel heard, and it needs to feel like it’s an individual approach and that it is collaborative and not “here is what we’re going to tell you to do”.

And similarly, clinicians expressed if no firmness was present, the young person would not feel contained as no limit setting would be present.

…you sometimes just have to have that stricter approach. But it’s coming from a place of love to try and make that change happen, otherwise anorexia just pushes back and knows, like, if there’s no sort of strict boundaries or strict things in place then I feel like it will just take any escape route it can to get away with things. So, like, they need that firmness and that harshness sometimes, but in a loving and empathetic way, it needs to be, obviously.

#### Relationships between parents

The relationship between the differing parents on the program was often described as creating a sense of *“solidarity”.* EDIPA holds a parent group twice weekly where parents can discuss challenges they are facing and the changes they have noticed in their young person. It was thought this shared experience with other parents in similar situations created hope and effectiveness for parents.

And parents who have felt stuck for so long, seeing themselves in other parents and family situations that are not that different. And getting hope that OK if we do this in this way, we might get there.

#### Relationships between parents and young people

Clinicians expressed the importance of targeting the relationships between parents and their child in treatment. When a strong relationship was present between the parents and young people, there was more understanding for the young person’s distress and insight into their pushback on meals. This understanding of the eating disorder and their child’s distress allowed more opportunity to settle the young person and elicit change in AN symptomology.

The treatment places a lot of strain on the family, so you know, if the cup is almost already overflowing, there’s not much more capacity to take on stress. So, if there’s strong relations and coping mechanisms within the family, there’s just more tolerance, more capacity to tolerate with the strains that it places on them. So, the parents, you know, are less likely to get strung out and are more likely to be able to kind of keep their cool in those moments, which provides like, a calming effect on the young person to be able to finish the meal.

However, if this relationship was not present, there would be less chance of change occurring because the AN had found an opportunity to take charge and limit refeeding and thus an important target of treatment to strengthen the relationship.

Because if you know if some young person is seeing that the illness is, you know, is scaring the parents or is able to bully the parents and then it gives it, you know, a bigger voice and anorexia is just very good at finding cracks.

#### Relationships between young people

The relationship between young people on the program was described as having the capacity to be both helpful and unhelpful. Staff described that there could be a sense of comradery between patients with young people being supportive of each other, especially when doing hard things together, such as meals. Young people in the program were perceived to experience a sense of connection, shared understanding, and increased empathy with each other through the sharing of their lived experiences. When young people are at different stages in the program with some young people moving forward towards recovery this was seen as potentially increasing hope and openness for recovery.

Seeing other young people make psychological/functional progress can help encourage young people/families working towards functional gains e.g. return to school.

Galvanising…the milieu [group] pulls stuck individuals forward.

Staff reflected that the group milieu between patients was highly valued by staff as a change agent and identified that considerable clinical thinking went into maintaining a helpful and constructive group milieu. They identified that the young people are highly sensitive to the group dynamics and that these can also be unhelpful. For instance, when young people tended to band together with each other in unhelpful ways, rather than receive support from the clinicians or their parents, it increased the risk of unhelpful conversations taking place, which ultimately maintain the AN (e.g., falsifying weight, purging strategies).

Sometimes the young person or young people can impact on each other and that can really, really prevent change. Just like them banding together with each other rather than, it’s not that we want them to band together with us, but more leaning on each other instead of us…or leaning on their parents, which would be more helpful, I think, and more appropriate in making change happen.

…there might be discussions around compensatory behaviours like exercising or how to falsify weight or how to purge, in sort of illness promoting discussions that I think the eating disorder is quite receptive to.

### Theme 2b: team mutuality and collaboration

The clinicians described the clinical team as a cohesive network in which different disciplines can mutually share a feeling, action or relationship, and collaborate to create a holistic understanding of the young person and their family. It was thought that the team as a whole was *“more than the sum of its parts”* because different perspectives were always present to provide nuance and richness to the treatment plan and the family’s treatment goals. In fact, there was a sense that change would not be possible if all the parts of EDIPA were not interconnected and working together.

Yeah, ’cause, if you think that of all the different components…we’ve got the therapy groups, we’ve got the parent groups, we’ve got family therapy, the medical, psychiatry… all those things are really important, but in and of themselves, I don’t think will yield change. So, it’s all the things together, but it’s also the way in which they are held by the team. So, I think if you took all those things out and you didn’t have a cohesive team that understood the assignment, it doesn’t work. So, the relational aspects of this [at EDIPA] are crucial.

*“I also believe that we have like a reasonable safety where people have the freedom to express their opinions. You know the collaboration within the teamwork. So*,* I think all these things*,* because if you professionally feel safe*,* you feel empowered*,* you feel safe to share your concerns”.*

### Relational containment

As with structural containment, it was understood that the aforementioned two themes (Relationships and Team Mutuality and Collaboration) combined to create relational containment. This was understood as the internal social factors (e.g., relationships between the staff and parents, relationships between the staff), within the structure of the program that support and create relational safety for the families.

## The combination of relational and structural containment

When combined, relational and structural containment were understood to facilitate an individual process within the family system at both the parent and young person level. For parents, parental effectiveness was enhanced through (1) increased insight in understanding the nature of AN and subsequently what was required in treatment; and (2) moving into a more active, change-oriented position in the treatment via making and supporting behavioural changes. Parental effectiveness was understood as parents feeling both effective and confident in their ability to promote change in themselves and their young person. For example, parents were more able to deliver eating disorder treatment such as supporting their child to eat a wider range of foods of sufficient volume, managing and reduce unhelpful eating disorder behaviours, such as compulsive exercise, purging etc. and generally more effectively managing their child’s distress. Clinicians often expressed that the greatest likelihood of recovery occurs when parents are supported to become *“the primary agents”* of change for their young person.

For young people, the relational focus and structure of the program were thought to not only settle young people, but help them meaningfully engage in the program, which in turn supported parental effectiveness. This process was perceived to generate positive change for the young person and their family and to contribute more broadly to attunement and family connection.

### Levels of relational and structural containment

**Fig. 2:**
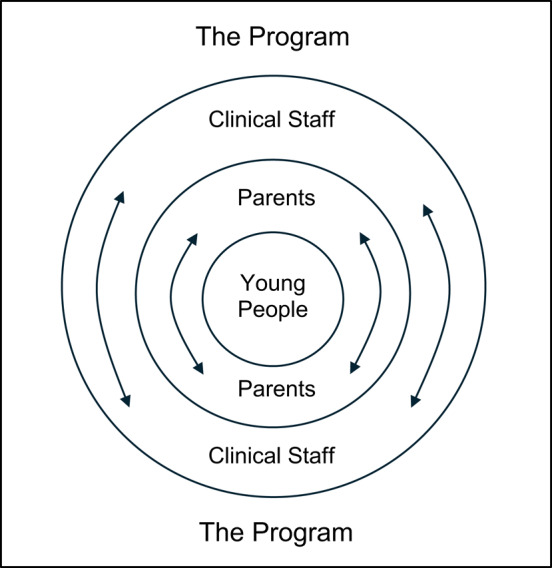
‘Levels’ of relational and structural containment thought to provide a wrap-around approach to support parents in leading the change in their young person. The program structure of EDIPA was thought to contain the clinical staff and families, clinical staff were thought to contain themselves and families, and parents were thought to contain themselves and their young person

Clinicians also expressed ideas about different ‘levels’ of relational and structural containment. One level was understood to be the program structure of EDIPA as a whole, which contained the clinicians as well as the families. The clinicians also contain themselves and the families, and the parents contain themselves and their young person (see Fig. 2).

There’s containment on so many levels, there’s like the young person feeling contained by like the team and like by their parents, there’s like the whole family feeling contained by the team. There’s the team containing the team in the context.

These levels of containment were understood as providing a wrap-around approach where clinicians support each other and feel supported by the program structure. The clinicians then support parents to settle and engage their young person and create behavioural change in their family.

But it’s the way our program is designed to just, you know, I think support, guide, direct in a way but also as I said, sort of empower the parents to make the change, I think and feel supported to make those changes.

## Discussion

The aim of the present study was to explore how change was perceived to occur within an adolescent eating disorder Day Program from the perspective of a team of clinicians, a hitherto neglected perspective in the literature. The combination of a treatment response that was both structurally and relationally containing for families was perceived by clinicians as the leading mechanisms in supporting an effective treatment context. These mechanisms were understood as strengthening the family system through a bi-directional relationship between increased parental effectiveness and settling and engagement of the young person.

Relational containment within the family system has been hypothesised as an important change mechanism in outpatient FBT [22]. While Wallis and colleagues described elements of structure, a care team, therapy appointment frequency, medical appointments within the original relational containment concept, the data in the current study suggests the additional aspects of structural *and* relational containment are important in leading to positive treatment changes in more intensive levels of care. It is possible that families needing more intensive levels of care require both processes of containment because they present with an increased complexity, compared to young people attending outpatient FBT. This idea is supported by recent qualitative findings reported from young people and their families in another Day Program setting [5]. The findings of the present study complement the findings of Colla and colleagues who found that the process of containment was linked to the program’s structure and authority, while the process of connection referred to the families’ relationships to staff, peers and each other, suggesting that both structural and relational factors are necessary and work in tandem to create change in the Day Program setting.

While the importance of structural and relational containment is understood as important for positive change in the current study, an additional finding was that the length of an admission might be crucial to these processes working effectively. Such containment might turn into dependence if held by teams for too long. Thus, clinicians holding these ideas might need to consider how to sufficiently support families with intensive but short-term supports with a view of gradually reducing supports to work towards greater independence. Discovering a ‘sweet spot’ between dependence and independence is likely to be a consistent process issue for teams with each individual family in such programs.

Day Programs likely hold a unique possibility of creating such containment because of the day-to-day nature of the program and the subsequent possibility of creating relationships and rigid program structure. For clinicians not working in such intensive settings, how to create a more structurally and relationally containing treatment response might be useful, particularly for families who are struggling with a positive treatment response. Determining which families might present with high or low containment needs could be a focus of future research.

Relational and structural containment embedded in the program model were also understood as containing the clinical staff, which in turn allowed the staff to contain the parents, and the parents their child. The findings from the current study thus extend on existing ideas regarding containment and expand the containment boundaries to include the clinical team. In this way, we are understanding these processes of containment as layers of safety. The nested structure of this suggests a sense of unity and connection between different parts of the whole, ultimately resulting in relational safety in which the hard parts of treatment can be held. Consequently, the structure and relational experience of the Day Program might replicate a secure base for families – acting as a safe base or *‘meta parents’* – so that families can tolerate the challenges of treatment, such as supporting young people to eat feared foods, challenge eating disorder behaviours, and tolerate distress in ways that feel safe. This might be particularly important if the stress of a serious eating disorder means that such containment or consistency is not possible at home. Additionally, staff create safety with each other, and the program structure at large potentially allowing them to better support parents and young people. These findings potentially highlight the importance of considering how to create an effective systemic treatment context.

These findings also speak to the importance of relationship safety as a foundation for working with families experiencing significant distress. Consistent with attachment theory [23, 24], adolescents need their caregivers to turn to them and respond in predictable and attuned ways in the face of serious difficulty to maintain safety. In a parallel way, the clinical team also need to be a secure base for the families with a similar predictability and attunement. A kind of transitional object [25, 26] for the uncertainty until a new safer, more regulated, forward moving situation emerges. Security and safety have important consequences for both regulating a system’s emotional tone and reducing anxiety levels [27]. As such, shifts that support effective problem solving and the creation of a positive social learning environment between people who are relevant to each other is important [28, 29]. Without a secure base ‘hard things’ become impossible, and support offered can feel like it is ‘missing the mark’. Given the intense and distressing nature of AN [30, 31], and the stress and impact placed on families [32], the need for a secure base in which to receive the effectiveness of treatment is understandable.

The potential importance of team mutuality and team processes is another novel finding from the present study. How to create safety and containment within a clinical team was not a focus of the current study but could be a focus of future research. Participants in the current study emphasised the importance of clinicians from all disciplines (medical, psychological, nursing, dietetics etc.) collaborating to drive change resulting in a less hierarchical and top-down approach. The findings highlight the importance of the clinical team working cohesively, and its role in creating change for families. This is in line with a broader recognition in medicine and associated fields that interdisciplinary (shared decision making between professionals) ways of working lead to better patient outcomes and also positive interpersonal relations for clinicians [33, 34]. How to establish and maintain effective interdisciplinary care and the associated safety is an important consideration, particularly given the intensity of eating disorder treatment, potential stress on clinicians, and flow-on effects to families and young people. Building clinical team safety into the model of care for intensive eating disorder services might be an important consideration for future service designs.

For most families, such levels of containment will not be necessary for effective treatment outcomes, as evidenced in the broad usefulness of FBT [8, 35–37]. For families where there is a higher level of complexity or higher strength of illness, how we create a context and supportive environment that allows effective treatment to occur rather than changing treatment approaches, should be considered. Importantly, the concepts reported in this study suggest that containment alone is not sufficient; rather it creates a treatment context that allows families to effectively engage in evidenced-based approaches.

The findings of the current study emphasised therapeutic processes more than content. While participants in the current study reflected on family therapy concepts in their responses, there was little focus on treatment content with much greater emphasis on therapeutic processes and treatment delivery. These findings possibly speak to the importance of therapeutic stance when greater treatment intensity is required, with services potentially needing to emphasise *how* treatment is delivered as a critical component of a model of care alongside evidenced-based treatments. This might be particularly important for families who are needing a higher level of care and have already experienced poor response in standard evidence-based treatments.

## Strengths and limitations

A strength of the present study is the recruitment of all clinical staff who worked at the study site. While the sample size was somewhat small, perspectives were gathered from a diversity of clinical backgrounds (e.g., from dietetics, psychiatry, nursing, psychology and social work) which added nuance and richness to focus groups. Interestingly, no significant differences in findings between the disciplines were found. Given the interviews were conducted with one team, and the program’s emphasis on reflective practices, it is likely this alignment in responses is due to the clinical staff regularly learning from each other and combining ideas together. Consequently, findings of this study might not be generalisable to other clinical settings. However, a specific aim of the current study was to use qualitative research methods to overt the clinical thinking of a team of clinicians working in this speciality area.

The present study extends on similar qualitative research that has explored how change occurs from the perspectives of young people and parents [5]. In family therapy, second order cybernetics recognises the clinician’s role in shaping outcomes and the co-creation of meaning [38]. Thus, the addition of clinician perspectives was needed for a full systemic understanding of change processes. Future qualitative research could encompass all perspectives (parents, young people, and clinicians) into one analysis and explore if findings from the perspective of clinicians are similarly held by parents and young people who attend such services.

## Conclusions

The present study sought to explore how changes occur within an adolescent eating disorder Day Program from the perspective of a team of speciality clinicians. Change was perceived to occur through the strengthening of the family system, driven by processes of structural and relational containment. These processes are facilitated through mechanisms of structure, predictability, intensity, tailored and responsive treatment, relationships and team mutuality and collaboration. The findings underscore the significance of safety and containment for young people and parents participating in an intensive eating disorder Day Program, as a means of supporting parental effectiveness and engaging and settling young people so that effective treatment can occur. Relational and structural containment for families, young people, as well as the clinicians delivering treatment, should be considered as important considerations for future service design and delivery.

## Data Availability

The datasets used and/or analysed during the current study are available from the corresponding author on reasonable request.

## Abbreviations

AN: Anorexia Nervosa
FBT: Family Based Therapy
CBT: Cognitive-Behavioural Therapy
EDIPA: Eating Disorder Intensive Program for Adolescents
SCHN: Sydney Children’s Hospital’s Network
CPI: Coordinating Principal Investigator
